# Occurence of a round window membrane rupture in patients with sudden sensorineural hearing loss

**DOI:** 10.1186/1472-6815-12-14

**Published:** 2012-11-29

**Authors:** Frank Haubner, Christian Rohrmeier, Christoph Koch, Veronika Vielsmeier, Jürgen Strutz, Tobias Kleinjung

**Affiliations:** 1 Department of Otorhinolaryngology, University of Regensburg, Franz-Josef-Strauß-Allee 11, Regensburg 93053, Germany; 2 Department of Otorhinolaryngology, University of Zurich, Zurich, Switzerland

## Abstract

**Background:**

Aim of the present study was to evaluate the occurence of a round window membrane rupture and the effects of hearing restoration after exploratory tympanotomy and sealing of the round window (niche) in patients with unilateral sudden deafness.

**Methods:**

Retrospective analysis of patients’ charts in a tertiary referral center. Charts of 69 patients with sudden deafness followed by exploratory tympanotomy were retrospectively analyzed. Pure-tone audiometry data before and after tympanotomy were compared to determine the outcome of hearing recovery. The postoperative hearing test values were documented 3 weeks after tympanotomy. All surgical reports were reviewed with regard to the surgical technique performed and the intraoperative findings.

**Results:**

18.8% of the patients revealed a visible perilymphatic fistula in the round window niche. 89.8% of the patients reported no typical history for a round window membrane rupture. All patients were treated with an exploratory tympanotomy under local anesthesia and an intravenous corticosteroid treatment regimen. The majority of the surgeons used a fat plomb to cover the round window. Postoperative hearing was significantly improved compared to the preoperative hearing test data. No patient showed a worsened hearing curve after the treatment.

**Conclusion:**

Most patients suffering from unilateral sudden deafness had no visible perilymphatic fistula. In our study population, the majority of patients reported no typical history of a pressure elevation in the inner ear. Exploratory tympanotomy is a safe procedure that may support hearing recovery in patients with sudden deafness in addition to the established treatment regimen including high-dose steroids.

## Background

Considerable interest has been shown in sudden sensorineural deafness in recent years, especially since Simmons postulated that this could be caused by mechanical disruption of the membranes in the inner ear [1,2]. The round window membrane is the only soft tissue barrier between the middle and the inner ear. The round window membrane consists of 3 basic layers and is a semipermeable membrane. The mean thickness of the round window membrane of normal temporal bones was found to be about 67 μm [3].

A perilymphatic fistula in the middle ear caused by a deficient round window membrane (RWM) can result in the symptoms of hearing loss, tinnitus and vertigo, either solely or in combination. Many of these patients were supposed to have a predisposing incident with consecutive pressure elevation in the inner ear. Otic barotrauma is well-documented in air travel [4] and diving accidents [5], but there are also patients suffering from sudden deafness related to the rupture of theRWM without any barotrauma.

While it appears that vertigo uniformly responds very satisfactorily to operative treatment, the improvement in hearing loss and tinnitus is more difficult to predict [6,7]. The degree of hearing loss varies in patients with a perilymphatic fistula, and the cause of this variability is not well understood. One factor might be the cochlear blood flow, which seems to decrease after a RWM rupture [8].

RWM defects are difficult to diagnose and no consensus of treatment has been reached [9]. There are different reports of intraoperative findings [10,11] and diagnostic instruments to visualize a perilymphatic fistula [10]. Many institutions recommend the exploratory tympanotomy, including an obliteration of the round window niche under local anesthesia [12,13].

Aim of our study was to determine the occurence of a RWM rupture in patients suffering from sudden sensorineural hearing loss. The outcome and safety of the exploratory tympanotomy, as well as the material used to seal the round window, were analyzed.

## Methods

Clinical charts of 69 patients with a history of unilateral sudden deafness between 2004 and 2011 were included in the study. Ethics was obtained for the entire study by the Institutional Review Board of the University of Regensburg (11-101-0232).

Sudden deafness was defined as sensorineural hearing loss of more than 50 dB HL in three or more contiguous frequencies in pure-tone audiometry as compared to the normal hearing ear. There exist several studies concering the evaluation of the outcome after exploratory tympanotomy. Ul-Mulk et al. [14] included patients with SSNHL of more than 40 dB HL. Other authors report about 60 dB HL as definition for a unilateral deafness [15]. Therefore we decided for more than 50 dB HL in at least three contiguous frequencies as compared to the contralateral ear to define a severe hearing loss and as indication for an exploratory tympanotomy.

Pure-tone audiometry (PTA) was used to calculate the thresholds. To calculate the out-of-limits the PTA value was set to 120 dB hearing loss. The improvement was calculated with respect to this value in each frequency.

There was no evaluation of PTA values prior to the event of sudden deafness. All patients were treated simultaneously with high-dose steroids. The exploratory tympanotomy was performed within 48 h after diagnosing the severe hearing loss if no improvement of hearing was observed.

Clinical presentation including vertigo and tinnitus was documented, but the indication for surgery in all cases was the hearing loss. The complete medical history including noise exposure was reviewed.

The surgical reports with respect to the intraoperative findings and the detailed technique of round window obliteration were analyzed.

All patients obtained an exploratory tympanotomy under local anesthesia and received a sealing of the round window niche. The procedures were done via an endaural approach to the middle ear after raising a tympanomeatal flap. Using the operation microscope, the chorda tympani was preserved and the auditory ossicles were inspected. In some cases it was necessary to remove parts of the lateral attic wall to obtain a complete overview of the stapes foot plate and the round window niche. The round window membrane is mostly hidden in the depth of the round window niche. That is why false membranes und mucosal folds were removed by the surgeons routinely. Drilling bony overhangs was not part of the procedure regulary.

A clearly visible rupture of the RWM with persisting fluid in the round window niche after suctioning was the criterion for a “definite fistula”. Due to the difficulties to categorize a definite fistula in case of missing overview of the complete RWM, the category of “doubtful fistula” was integrated to the study according to Maier [15] for subjects with persisting fluid in the round window niche instead of suctioning.

All procedures were done by three experienced otological surgeons who used the criteria mentioned above for their diagnosis.

Statistical analysis was done using the Wilcoxon-Test for the PTA data. Correlations were determined by the Chi-square test.

## Results

### Patients’ characteristics

69 charts of patients with a suspected round window membrane rupture were analyzed between 2004 and 2011. The mean age was 56.9 years (17 to 92 years). 30 females and 39 males were included into the study. 56.5% of the cases showed a hearing loss on the left side. 43.5% of the patients had impaired hearing on the right ear. With respect to the whole study population, 44.9% reported vertigo and 50.7% reported a new manifestation of tinnitus.

89.8% percent of the patients had no typical history for a round window membrane rupture. 10.2% of the patients reported a typical history with physical exercise (3/69), diving (1/69), head trauma (2/69) or noise exposure (1/69) coinciding with the start of complaints (Table 1).

**Table 1:** **Characteristics of patients and their neurotological findings **(**n**=**69**)

**Patient**	**Typical history**	**Side of hearing loss**	**Vertigo**	**Tinnitus**
1	No	Left	Yes	Yes
2	No	Right	no	Yes
3	No	Left	Yes	No
4	No	Left	No	Yes
5	No	Right	No	Yes
6	Head trauma	Right	No	Yes
7	No	Right	Yes	No
8	Physical Exercise	Right	No	Yes
9	Noise	Right	Yes	No
10	Diving	Left	No	Yes
11	No	Right	Yes	Yes
12	No	Left	No	Yes
13	No	Right	No	Yes
14	No	Left	Yes	Yes
15	Physical Exercise	Left	No	No
16	No	Left	Yes	No
17	No	Left	No	No
18	No	Right	No	No
19	No	Right	No	No
20	No	Left	Yes	No
21	No	Right	Yes	Yes
22	No	Left	No	Yes
23	No	Right	No	Yes
24	No	Left	Yes	No
25	No	Right	No	No
26	No	Left	No	Yes
27	No	Right	No	No
28	No	Left	Yes	Yes
29	No	Left	Yes	No
30	No	Left	No	No
31	No	Left	Yes	Yes
32	No	Left	No	No
33	No	Right	Yes	No
34	No	Left	No	Yes
35	No	Left	No	Yes
36	No	Right	No	No
37	No	Left	Yes	Yes
38	No	Left	Yes	No
39	No	Left	No	No
40	No	Left	Yes	No
41	No	Left	No	No
42	No	Right	Yes	Yes
43	No	Left	No	No
44	No	Left	Yes	Yes
45	No	Left	Yes	No
46	No	Left	No	Yes
47	No	Right	Yes	Yes
48	No	Left	No	Yes
49	No	Right	Yes	No
50	No	Right	No	Yes
51	No	Right	No	Yes
52	No	Left	Yes	No
53	No	Right	No	No
54	No	Left	Yes	Yes
55	Physical Exercise	Left	No	Yes
56	No	Right	No	Yes
57	No	Left	Yes	No
58	No	Right	Yes	Yes
59	No	Right	Yes	No
60	No	Right	No	No
61	No	Left	Yes	No
62	No	Left	No	Yes
63	No	Right	Yes	No
64	No	Left	No	No
65	No	Right	No	Yes
66	No	Left	No	No
67	No	Right	No	Yes
68	No	Left	Yes	No
69	Head trauma	Right	Yes	Yes

### Correlation analyses

There was a significant correlation (p=0.001) of the age and the amount of the preoperative hearing loss on the affected ear (pearson coefficient=0.467), as well as of the age and the postoperative hearing on the affected ear (pearson coefficient=0.363).

There was no statistically significant association between the medical history for a round window membrane rupture and the intraoperative finding (p=0.3). There was no significant correlation of the history of the patient and the diagnosis of tinnitus (p=0.4) or vertigo (p=0.4) preoperatively. There was also no association between the diagnosis of tinnitus (p=0.6) or vertigo (p=0.09) and the intraoperative finding of a perilymphatic fistula.

### Analysis of intraoperative findings and surgical techniques

**Figure 1:**
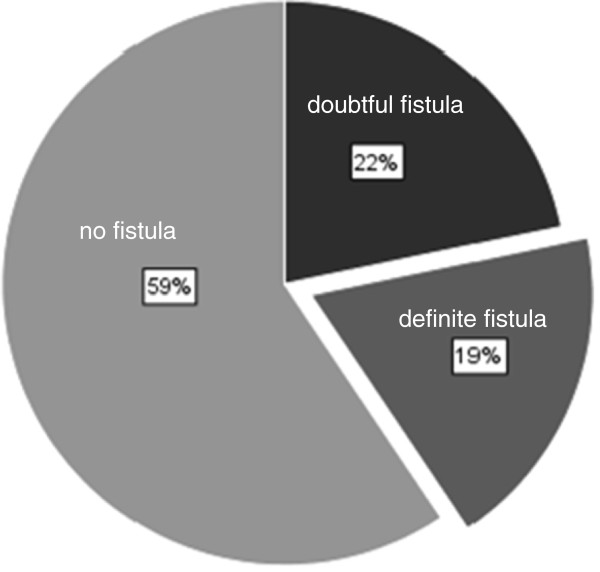
Statistical evaluation of intraoperative findings with respect to the operation reports of 69 patients.

The evaluation of the surgical reports revealed no fistula in 59.4% of the cases, a definite sign of round window membrane rupture with clear fluid in 18.8% and a doubtful perilymphatic fistula in 21.7% of the patients (Figure 1). All patients obtained an exploratory tympanotomy under local anesthesia and received a sealing of the round window niche. The procedures were done via an endaural approach to the middle ear after raising a tympanomeatal flap. Using the operation microscope, the chorda tympany was preserved and the auditory ossicles were inspected. In some cases it was necessary to remove parts of the lateral attic wall to obtain a complete overview of the stapes foot plate and the round window niche. 81.2% of the patients were treated with a fat seal and 11.5% with a sealing built of fibrous soft tissue like fascia or perichondrium. In the remaining 5 patients, combinations of soft tissue and fat as well as combinations with fibrous glue were used to cover the round window membrane (Table 2). The three patients covered with fibrous glue showed an improvement of hearing of more than 20 dB. Due to the small sample size there was no significant correlation between the different techniques and the outcome.

**Table 2:** **Intraoperative findings according to the categories**: **fistula**, **no fistula and doubtful fistula**

**Patient**	**Intraop finding**	**Material**	**Improvement of hearing**
			
1	fistula	fat	< 20 dB
2	no fistula	fat	> 20 dB
3	doubtful fistula	fat	> 20 dB
4	doubtful fistula	fat	no change
5	no fistula	fat	> 20 dB
6	fistula	fat	< 20 dB
7	doubtful fistula	fat	> 20 dB
8	fistula	fat	< 20 dB
9	fistula	fat	< 20 dB
10	doubtful fistula	fat	> 20 dB
11	doubtful fistula	fat	> 20 dB
12	no fistula	soft tissue	> 20 dB
13	no fistula	fat	> 20 dB
14	fistula	fat	< 20 dB
15	no fistula	fat	< 20 dB
16	doubtful fistula	fat	> 20 dB
17	no fistula	soft tissue and fibrin glue	> 20 dB
18	no fistula	fat	> 20 dB
19	doubtful fistula	fat	> 20 dB
20	doubtful fistula	fat	< 20 dB
21	no fistula	fat	< 20 dB
22	doubtful fistula	fat	no change
23	no fistula	fat	> 20 dB
24	no fistula	fat	> 20 dB
25	no fistula	soft tissue	< 20 dB
26	no fistula	fat	< 20 dB
27	doubtful fistula	fat	< 20 dB
28	doubtful fistula	fat	no change
29	doubtful fistula	fat	> 20 dB
30	doubtful fistula	soft tissue	> 20 dB
31	no fistula	fat	> 20 dB
32	no fistula	soft tissue	> 20 dB
33	fistula	fat	> 20 dB
34	no fistula	fat	< 20 dB
35	no fistula	fat	no change
36	no fistula	fat	< 20 dB
37	fistula	fat	no change
38	no fistula	fat	< 20 dB
39	no fistula	fat	< 20 dB
40	no fistula	fat and soft tissue	< 20 dB
41	no fistula	fat	> 20 dB
42	no fistula	fat	< 20 dB
43	no fistula	fat	< 20 dB
44	no fistula	fat	no change
45	no fistula	fat	no change
46	no fistula	fat	> 20 dB
47	no fistula	fat	< 20 dB
48	no fistula	fat	> 20 dB
49	no fistula	fat and soft tissue	< 20 dB
50	no fistula	fat	no change
51	no fistula	soft tissue	> 20 dB
52	no fistula	fat	< 20 dB
53	no fistula	fat	< 20 dB
54	doubtful fistula	soft tissue	> 20 dB
55	no fistula	fat	< 20 dB
56	fistula	fat and fibrin glue	> 20 dB
57	fistula	fat	> 20 dB
58	no fistula	fat	> 20 dB
59	fistula	fat	> 20 dB
60	no fistula	fat	> 20 dB
61	no fistula	fat	> 20 dB
62	no fistula	soft tissue	no change
63	doubtful fistula	fat	> 20 dB
64	fistula	fat	> 20 dB
65	no fistula	soft tissue	> 20 dB
66	no fistula	fat	< 20 dB
67	fistula	fat and fibrin glue	> 20 dB
68	fistula	fat	> 20 dB
69	no fistula	fat	no change

Material used to cover the round window niche and postoperative hearing improvement (n=69).

All patients were simultaneously treated with intravenous steroids starting with 500 mg prednisone following a reduction scheme over 12 days.

### Postoperative evaluation (after 3 weeks)

**Figure 2:**
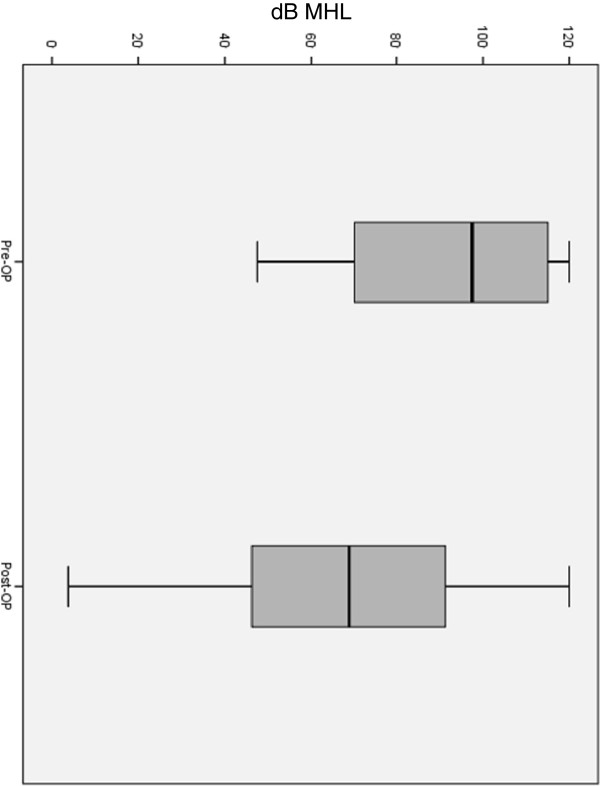
**Mean hearing loss (MHL) pre- and postoperatively: Mean values in dB HL were calculated for 500 Hz, 1000 Hz, 2000 Hz and 4000 Hz. **Box plots include median, 25%/75% percentile (boxes) and minimum/maximum values (whiskers). (n=69, p<.,001).

Hearing was improved by more than 20 dB in 43% of the cases. In 18 patients (26%) there was no improvement in hearing observed. 31% of the patients showed a minor improvement in hearing between 5 dB and 20 dB. Postoperative hearing was significantly improved as compared to the preoperative PTA data (Figure 2, Table 3).

**Table 3:** **Mean values of hearing loss **(**MHL**) **pre**- **and postoperatively with respect to the PTA values in 500 Hz**, **1 KHz**, **2 kHz**, **4 kHz**

	**500Hz**	**1KHz**	**2KHz**	**4KHz**
**MHL preop **(**dB HL**)	90,6	92,1	93,8	96,0
**MHL postop **(**dB HL**)	59,4	66,2	78,2	80,5

In the group of subjects with the intraoperative finding of a definite perilymphatic fistula, 7 patients (53%) showed an improvement in hearing of more than 20 dB postoperatively. The latter 10 subjects in this group revealed no change in hearing after the tympanotomy and none suffered from a worsening in hearing after the treatment. Bone and air conduction were evaluated 3 weeks after surgery. There was no worsening observed postoperatively. 17 patients (41%) with no fistula improved by more than 20 dB after the treatment. There was no significant difference in hearing recovery between the groups of definite, doubtful and no round window membrane rupture.

Complications related to the exploratory tympanotomy (e.g. facial nerve injury, change in sense of taste, persistent tympanic membrane perforation) could not be observed.

## Discussion

Sudden sensorineural hearing loss (SSNHL) is a severe impairment for the affected person. Over the past decades, no specific treatment regimen could be established [16]. One reason for that might be the fact that the majority of patients with an idiopathic sensorineural hearing loss show a high recovery rate even with no specific treatment [17]. Exploratory tympanotomy was often indicated for the management of SSNHL occurring in the context of head trauma, barotrauma and chronic otitis media with cholesteatoma and in patients with congenital inner-ear abnormalities [17,18]. Especially for patients after a diving accident, there is a clear indication for performing an exploratory tympanotomy [19]. But it remains unclear whether patients with no typical history for a round window membrane rupture benefit from this procedure. There are a few retrospective studies that analyzed hearing after tympanotomy [14,15,20]. Selmani et al., who performed an endosopic inspection of the middle ear cavity, observed no perilymphatic fistula in 265 cases of patients with SSNHL and vertigo [21]. With respect to the well-established microsurgery of the middle ear and the possible infection during a middle ear endoscopy, we prefer the better visualization of the round window membrane via the operation microscope. Reports of patients who underwent an exploratory tympanotomy in our institution due to sudden deafness over the last 6 years were retrospectively analyzed. There was only a minority of 10% of patients with a typical history of increased inner ear pressure with respect to the onset of symptoms. This is in contrast to Taylor et al., who reports 18 of 20 patients with a history of pressure elevation in context with the hearing loss [22]. A similar retrospective study by Maier et al. reports 22% of patients had a typical history of perilymph fistula [15]. We observed intraoperatively definite round window membrane ruptures in about 20% of the cases. This is in contrast to Arndt et al. [11], who documented a spontaneous perilymph fistula in 60% of their study population. Maier et al., who analyzed the same categories of intraoperative findings (fistula, no fistula and doubtful fistula), found a definite fistula in 35% and no fistula in 37% [15].

Assessment of perilymphatic fistulas remains a diagnostic problem. In our study we had a rate of 22% of doubtful fistulas. Visualizing the round window membrane often demands the removement of false membranes and bony ridges. Exact data on this technical detail were not possible to evaluate retrospectively in all cases. Indirect signs as the observation of persisting fluid in the round window niche were therefore considered as a criterion for the diagnosis of a doubtful perilymphatic fistula in the present study.

Whether other methods such as intrathecal fluorescein for perilymph staining are useful in this issue is still unclear [23]. Poe et al. analyzed the value of intravenous fluorescein applications in an animal model and concluded that the administered fluorescein causes dramatic fluorescence of vessels and transudates that may be interpreted falsely as fluorescence of perilymph [24].

Only 4 of our patients with a definite or doubtful fistula reported a typical history. One conclusion concerning this observation might be that the patients’ history does not predict the finding of an intraoperative round window membrane rupture. Thus the anamnesis concerning a predisposing incident is not a reliable indication for the surgery.

18 patients (26%) had no hearing improvement in the control examination after 3 weeks in our study population. This result is similar to Gedlicka et al. [20], who performed a retrospective study with 60 patients after tympanotomy and found no improvement in 33% of their patients.

There was a correlation between the age and the pure-tone audiometry values, which demonstrated that older people had a more severe impairment of hearing pre- and postoperatively. There was no correlation between the history of the patient, the intraoperative finding and the diagnosis of tinnitus or vertigo preoperatively. That means that we could not predict the finding of a perilymphatic fistula. The analysis of our pre- and postoperative pure tone audiometry values revealed significantly improved hearing postoperatively. Almost half of the patients had an improvement of their mean average hearing loss by more than 20 dB. This result might be due to tympanotomy and the covering of the round window niche. But we have to consider other possible effects: All patients were simultaneously treated with intravenous steroids starting with 500 mg prednisone following a reduction scheme over 12 days. Also placebo effects and a spontaneous recovery have to be mentioned and could not be excluded. There is also a possible bias because only patients who consented to perform surgery were included to this study. A generally accepted definition of what constitutes improvement or recovery after a SSNHL is not existingamong studies and reports. One interpretation is an improvement of 20 dB in pure-tone audiometry [25] as chosen in the present study. Other authors use an improvement of 30 dB as definition for a relevant hearing recovery [26].

With respect to these limitations, exploratory tympanotomy seems to be a safe procedure, because none of the patients suffered from any major complications or a worsening of hearing afterwards. Concerning the technical issues, most of the surgeons at our institution used fat sealing to cover the round window niche. A variety of other techniques including fat-fibrin-glue [9] and postauricular collagen tissue [27] is described in the literature.

The occurence of a round window membrane rupture in SSNHL patients was under 20% in our study population. The patients’ history did not predict the finding of an intraoperative round window membrane rupture. Thus the anamnesis concerning a predisposing incident is not a reliable indication for the surgery.

We found no correlation between the hearing recovery of SSNHL patients with and without perilymphatic leak. Nevertheless, exploratory tympanotomy is a safe procedure that might be a useful addition to high-dose steroids in severe cases of SSNHL. With respect to our results an exploratory tympanotomy should be considered for patients with no improvement of hearing within 48 h after treatment with high-dose steroids and a SSNHL of more than 50 dB HL in three contiguous frequencies. Further prospective studies that compare different treatment regimens are necessary to identify the benefit of an exploratory tympanotomy in patients with sudden deafness.

## Conclusion

Most patients suffering from unilateral sudden deafness had no visible perilymphatic fistula. In our study population, the majority of patients reported no typical history of a pressure elevation in the inner ear. Exploratory tympanotomy is a safe procedure that may support hearing recovery in patients with sudden deafness in addition to the established treatment regimen including high-dose steroids.

## Competing interests

There is no conflict of interest. The authors confirm that they do not have any financial relationship concerning this research.

## Authors’ contribution

FH and TK had the idea for the study and drafted the manuscript. CR, VV and CK perfomed the data collection. CK and FH peformed the statistical analysis and designed tables and figures. JS and TK contributed by supervision and administrative support. All authors read and approved the final manuscript.

## Pre-publication history

The pre-publication history for this paper can be accessed here:

http://www.biomedcentral.com/1472-6815/12/14/prepub

## References

[B1] Freeman P Rupture of the round window membrane Acta Otorhinolaryngol Belg 1975 29 5 783 794 1224962

[B2] Simmons FB Theory of membrane breaks in sudden hearing loss Arch Otolaryngol 1968 88 1 41 48 10.1001/archotol.1968.00770010043009 5660028

[B3] Yoda S Round window membrane in Meniere's disease: a human temporal bone study Otol Neurotol 2011 32 1 147 151 10.1097/MAO.0b013e318200a0e0 21131881

[B4] Mirza S Richardson H Otic barotrauma from air travel J Laryngol Otol 2005 119 5 366 370 15949100 10.1258/0022215053945723

[B5] Rozsasi A Sigg O Keck T Persistent inner ear injury after diving Otol Neurotol 2003 24 2 195 200 10.1097/00129492-200303000-00011 12621331

[B6] Tonkin JP Fagan P Rupture of the round window membrane J Laryngol Otol 1975 89 7 733 756 10.1017/S0022215100080944 1176821

[B7] Nedzelski JM Barber HO Round window fistula J Otolaryngol 1976 5 5 379 385 994277

[B8] Liu X Effects of round window membrane rupture on cochlear blood flow and endocochlear potential Nippon Jibiinkoka Gakkai Kaiho 2003 106 7 723 729 10.3950/jibiinkoka.106.723 12931639

[B9] Cole GG Validity of spontaneous perilymphatic fistula Am J Otol 1995 16 6 815 819 8572149

[B10] Kleemann D Rupture of the round window--detection with fluorescence endoscopy HNO 2001 49 2 89 92 10.1007/s001060050715 11270200

[B11] Arndt HJ Spontaneous perforation of the membrane of the round window--a major cause of sudden deafness? Laryngol Rhinol Otol (Stuttg) 1984 63 9 439 444 10.1055/s-2007-1008329 6492958

[B12] Mertens J Rudert H Sudden deafness caused by rupture of the round window membrane. Surgical indications, course and prognosis HNO 1986 34 8 320 324 2428778

[B13] Strohm M Lesions of round window membrane Laryngol Rhinol Otol (Stuttg) 1982 61 6 297 301 10.1055/s-2007-1008578 7121148

[B14] Ul-Mulk J Friis S Hahn CH Tympanotomy and sealing of the round window for treatment of sudden deafness Dan Med Bull 2011 58 5 A4276 21535987

[B15] Maier W Results of exploratory tympanotomy following sudden unilateral deafness and its effects on hearing restoration Ear Nose Throat J 2008 87 8 438 451 18712692

[B16] Conlin AE Parnes LS Treatment of sudden sensorineural hearing loss: I A systematic review. Arch Otolaryngol Head Neck Surg 2007 133 6 573 581 10.1001/archotol.133.6.573 17576908

[B17] Schreiber BE Sudden sensorineural hearing loss Lancet 2010 375 9721 1203 1211 10.1016/S0140-6736(09)62071-7 20362815

[B18] Lu Y Idiopathic perilymph fistula Zhonghua Er Bi Yan Hou Ke Za Zhi 1989 24 1 15 16 62 2641874

[B19] Bohm F Lessle M Round window membrane defect in divers Laryngorhinootologie 1999 78 4 169 175 10.1055/s-2007-996852 10407821

[B20] Gedlicka C Formanek M Ehrenberger K Analysis of 60 patients after tympanotomy and sealing of the round window membrane after acute unilateral sensorineural hearing loss Am J Otolaryngol 2009 30 3 157 161 10.1016/j.amjoto.2008.04.003 19410119

[B21] Selmani Z Role of transtympanic endoscopy of the middle ear in the diagnosis of perilymphatic fistula in patients with sensorineural hearing loss or vertigo ORL J Otorhinolaryngol Relat Spec 2002 64 5 301 306 10.1159/000066074 12417768

[B22] Taylor PH Bicknell PG Rupture of the round window membrane Ann Otol Rhinol Laryngol 1976 85 1 Pt 1 105 109 1259309 10.1177/000348947608500119

[B23] Gehrking E Intraoperative assessment of perilymphatic fistulas with intrathecal administration of fluorescein Laryngoscope 2002 112 9 1614 1618 10.1097/00005537-200209000-00016 12352674

[B24] Poe DS Intravenous fluorescein for detection of perilymphatic fistulas Am J Otol 1993 14 1 51 55 8424476

[B25] O'Malley MR Haynes DS Sudden hearing loss Otolaryngol Clin North Am 2008 41 3 633 649 x-xi 10.1016/j.otc.2008.01.009 18436003

[B26] Rauch SD Oral vs intratympanic corticosteroid therapy for idiopathic sudden sensorineural hearing loss: a randomized trial JAMA 2011 305 20 2071 2079 10.1001/jama.2011.679 21610239

[B27] Paparella MM Sudden deafness secondary to a middle ear/inner ear interaction: The implications of finding an "adhesive tent" during tympanotomy Ear Nose Throat J 2009 88 2 776 777 19224477

